# Expression of 8-oxoguanine DNA glycosylase (*Ogg1*) in mouse retina

**Published:** 2009-06-05

**Authors:** Karine Bigot, Julia Leemput, Monique Vacher, Anna Campalans, J. Pablo Radicella, Emmanuelle Lacassagne, Alexandra Provost, Christel Masson, Maurice Menasche, Marc Abitbol

**Affiliations:** 1 Université Paris-Descartes, CERTO, Centre de Recherche Thérapeutique en Ophtalmologie, Paris, France; 2 Commissariat à l’Energie Atomique, Institut de Radiobiologie Cellulaire et Moléculaire, Fontenay aux Roses, France; 3 Service d’Ophtalmologie du CHU Necker-Enfants–Malades, Paris, France

## Abstract

**Purpose:**

The retina is highly exposed to oxidative stress due to the high level of oxygen consumption in this tissue and its exposure to light. The main DNA base lesion generated by oxygen free radicals is 8-oxoguanine (8-oxoG). However, its presence in retinal cells and the mechanisms underlying its repair remain undetermined.

**Methods:**

8-oxoguanine DNA glycosylase (*Ogg1*) gene expression and messenger localization in adult mouse ocular tissues was analyzed by RT–PCR and in situ hybridization. Using immunohistochemistry, we determined the localization of Ogg1 protein and three base excision repair (BER) enzymes: apurinic/apyrimidic endonuclease (APE1), DNA polymerase β, and X-ray repair cross-complementation group 1 (XRCC1). Ogg1 and AP-lyase activities in the neuroretina were obtained using double-stranded oligonucleotides harboring either an 8-oxoG residue or a tetrahydrofuran.

**Results:**

We report here that 8-oxoG is abundant in the retina. Ogg1, the enzyme responsible for the recognition and excision of the oxidized base, is present in its active form and found mainly in ganglion cells and photoreceptor inner segments. We show that APE1 and DNA polymerase β, two BER proteins involved in 8-oxoG repair, are also present in these cells. The cellular distribution of these proteins was similar to that of Ogg1. XRRC1 is present in both inner nuclear and ganglion cells layers; however, this protein is absent from photoreceptor inner segments.

**Conclusions:**

This is the first study to demonstrate the presence of a functional 8-oxoG BER pathway in retinal neurons. The study of three BER proteins involved in 8-oxoG elimination demonstrates that XRCC1 localization differs from those of Ogg1, APE1, and DNA polymerase β. This result suggests that the elimination of 8-oxoG is coordinated through two pathways, which differ slightly according to the cellular localization of the abnormally oxidized guanine.

## Introduction

There is growing evidence to suggest that endogenously generated reactive oxygen species (ROS) contribute to aging, cancer development, and neurodegenerative diseases such as Alzheimer disease, Parkinson disease, or inherited retinal dystrophies ( [1]; for review, see [2]). In vivo, free radicals are generated continuously as oxygen metabolism is required for normal physiologic processes. Additional harmful free radical production is also triggered by a variety of environmental agents, such as ultraviolet (UV), ionizing radiation, or exposure to chemical oxidants. The excess production of free radicals is controlled by the antioxidant defense mechanisms (e.g., superoxide dismutase, glutathione peroxidases, catalases). However, ROS that escape theses defenses can diffuse and interact with several cellular macromolecules, including nucleic acids [3].

The retina is characterized by higher oxygen consumption and metabolic rates than in other tissues [4-6], including all other parts of the central nervous system (CNS). Photoreceptor cells, in particular, are the cell type that has the highest rate of oxygen consumption per milligram of tissue in the whole body of mammalian species. Most of the ATP produced in retinal neurons, as in all neurons of the CNS, is generated by the glucose oxidative phosphorylation pathway. This pathway generates ROS, such as superoxide and hydrogen peroxide. Furthermore, specific physiologic characteristics of the retina, such as its exposure to light, UV radiations and the phagocytosis of photoreceptor outer segments disks by retinal pigment epithelial cells, contribute also to an elevated production of ROS in this nervous tissue [7-10]. As neuronal cells are in a postmitotic state, accumulation of oxidative damage can significantly disrupt transcription or activate replication, leading to cellular dysfunction and apoptosis (for review, see [11]). To date, little is known about the recognition and repair of oxidative DNA lesions in retinal cells.

Among the various DNA oxidative lesions [12], 7,8-dihydro-8-oxoguanine (8-oxoG) is the most abundant oxidized base generated in vivo by various types of ROS. This aberrant base is a premutagenic lesion, inducing G-C to T-A transversions. In mammalian cells, 8-oxoG is specifically recognized and excised by 8-oxoG DNA glycosylase (Ogg1). This enzyme initiates the highly conserved base excision repair (BER) pathway, the main responsible pathway for repair of small chemical alterations in DNA. Following the removal of the oxidized base by Ogg1, the abasic site is cleaved by an apurinic/apyrimidinic endonuclease (APE1), leaving a 5′-deoxiribose phosphate residue; this residue is removed by the AP-lyase activity of DNA polymerase β, which then also inserts a nucleotide. Finally, DNA Ligase III seals the repaired DNA strand. Other accessory factors such as X-ray repair cross-complementation group 1 (XRCC1) are also involved in this process [13].

In this study, we analyzed the presence, levels and activity of proteins involved in the BER of 8-oxoG in ocular tissues and particularly in the retina. We showed that Ogg1, the enzyme responsible for the recognition and excision of the 8-oxoG, is present at high levels in neuroretina and non-neuronal cells of the eye and that 8-oxoG DNA-glycosylase activity can be detected in these cells. We also found that the BER proteins, involved in the repair of 8-oxoG, were present in the retina with a cellular distribution similar to that of Ogg1.

## Methods

### Animals

Animals used for experiments were handled in strict accordance with the Association for Research in Vision Ophthalmology Statement on the Use of Animals in Ophthalmic and Vision Research. C57BL/6J (Elevage Janvier, Le Genest-Saint-Isle, France) and Ogg1-deficient mice (gift from Eliette Touati, Institut Pasteur, Paris, France with agreement of Arne Klungland, University of Oslo, Oslo, Norway) [14] were housed in a conventional temperature-controlled room (21 °C), exposed to a daily 12 h period of light, and fed ad libitum with a balanced diet determined by the Jackson laboratory for the C57BL6/J mouse strain. The experiments were performed on 2-month-old mice.

### Tissue samples

All mice were euthanized by cervical elongation. Brain and eyecups were rapidly removed. The neuroretina was removed from the eyecup. Tissues were frozen in liquid nitrogen and stored at −80 °C until further use. Enucleated eyes from adult mice were immediately fixed in 4% paraformaldehyde (PFA) for 16 h at 4 °C then embedded in paraffin for in situ hybridization and immunochemistry experiments. Next 5 μm-thick sagittal sections were cut using a microtome (HM355, Microm microtec, Francheville, France).

### RNA analysis

#### Reverse transcription

Total RNA was extracted using TRIzol® reagent (Invitrogen, Cergy-Pontoise, France) from mice adult neuroretina (n=3 for semiquantitative PCR and n=5 for quantitative PCR), forebrain (n=3), cerebellum (n=3), testis (n=5), and liver (n=5) according to the manufacturer’s instructions. Next, 1 µl aliquots of RNA were reverse transcribed, using SuperScript^TM^ II RNase H^-^ Reverse Transcriptase (Invitrogen, Cergy-Pontoise, France) in the presence of oligo(dT)_12–18_ primer.

#### Semiquantitative PCR

Two μl of cDNAs from neuroretina, forebrain, and cerebellum were amplified in a final volume of 25 μl of a PCR buffer constituted by 2 mM Tris/HCl and 1.5 mM MgCl_2_ and containing 0.2 μM of each primer, 0.2 mM of each dNTP (Promega, Charbonnières-les-Bains, France) and 0.5 U Taq DNA polymerase (Invitrogen, Cergy-Pontoise, France). Primers sequences and sizes of the PCR products obtained are described in Table 1. PCR amplification products were resolved by electrophoresis in a 1% (w/v) agarose gel and visualized by ethidium bromide staining under UV light.

**Table 1:** Details of primers and sizes of the amplified products.

Amplified cDNA	Primer sequences (5′-3′)	Size of PCR products (bp)
*Ogg1*	F: GATTGGACAGTGCCGTAA	400
	R: GGAAGTGGGAGTCTACAG	
*DNA polymerase β*	F: CATCAATTTCCTGACTCGAG	693
	R: TAGCGCCACTGGATGTAATC	
*APE1*	F: CTAAGGGCTTTCGTCACAGC	446
	R: GAGACTTTTAGCGGGCACTG	
*XRCC1*	F: CAGACAGCACACATCTCATC	418
	R: ACCCTCCTCAGTTCATCCT	
*Cyclophilin A*	F: TGGTCAACCCCACCGTGTTCTTCG	311
	R: TCCAGCATTTGCCATGGACAAGA	

#### Quantitative PCR

Real-time PCR was performed using the 2X Power SYBR-Green PCR Master Mix (Applied Biosystem, Courtaboeuf, France) on a final volume of 25 µl containing 50 ng of cDNA (neuroretina, liver, and testis) and 100 nM (Ogg1) or 20 nM (cyclophilin A) of forward and reverse primers. For determination of the initial relative quantity of cDNA, samples were amplified with *Ogg1* primers (5′-GAT TGG ACA GTG CCG TAA-3′ and 5′-GGA AGT GGG AGT CTA CAG-3′) and *cyclophilin A* primers (5′-TGG TCA ACC CCA CCG TGT TCT TCG-3′ and 5′-TCC AGC ATT TGC CAT GGA CAA GA-3′). *Cyclophilin A* was used as internal standard [15,16]. The cycling conditions comprised 2 min at 50 °C, 10 min polymerase activation at 95 °C and 40 cycles at 95°c for 15 s and 60 °C for 1 min. Reactions were run on an ABI Prism 7000 real time PCR machine; melt curves analyses were performed for all genes, and the specificity as well as integrity of the PCR products were confirmed by the presence of a single peak. The results were analyzed with the integrated 7000 system SDS software, and relative expression of *Ogg1* was calculated from the difference in cycle time of internal controls compared to the target mRNA. Data are expressed in the graphics as fold-expression ratio of normalized target gene, plus or minus standard error of the mean (SEM), according to the software results.

### In situ hybridization

Sense and antisense riboprobes were synthesized using a PCR-based in situ hybridization technique as previously described [17,18]. Briefly, PCR was performed using *Ogg1* gene-specific primers encompassing a T7 RNA polymerase binding site. Purified PCR products were then used for transcription reactions with T7 forward and reverse primers and T7 RNA polymerase to generate digoxigenin-conjugated sense and antisense *Ogg1* cRNAs. Sections were deparaffinized by incubation in xylene and rehydrated through a graded series of alcohol solutions, and in situ hybridization was performed [19]. Next, 150 ng of sense or antisense RNA probes were diluted in 150 μl the mRNA hybridization milieu (HIS hybridization solution, Dako, Trappes, France) and incubated with sections overnight at 62 °C in a humidified chamber. After three washes of 30 min at 60 °C with 1X Stringent Wash Concentrate (Dako), sections were incubated with 1:500 alkaline phosphatase-coupled anti-DIG antibody in antibody diluent (Dako) for 1 h at room temperature, then with substrates BCIP/NBT. Stained tissue sections were mounted with Aquatex (PolyLabo, Strasbourg, France).

### Immunohistochemistry

Paraffin was removed in xylene and sections were rehydrated and incubated for 20 min in 1X citrate buffer in a microwave at 500 W. Endogenous peroxidase activity was quenched by pretreatment with 3% H_2_O_2_ for 10 min. Sections were then treated with 0.3% Triton in 1X PBS (137 mM NaCl; 2.7 mM KCl; 4.3 mM Na_2_HPO_4_; 1.47 mM KH_2_PO_4_; pH 7.4) for 5 min. Sections were incubated overnight with a primary antibody at +4 °C. Control tissues were treated in the same way, but without antibody, to confirm that staining was specific to the antigen tested. The following antibodies were used in this study: 1:40 mouse anti-8-oxoguanine antibody (Gentaur, N45.1, Brussels, Belgium), 1:800 rabbit anti-human Ogg1 antibody (Novus Biologicals, Interchim, Montlucon, France), 1:800 rabbit anti-human APE1 antibody (provided by Ian Hickson, University of Oxford, Oxford, UK), 1:4,000 rabbit anti-full length rat polymerase β antibody (provided provided by Samuel H. Wilson, National Institutes of Health, Bethesda, MD) and 1:50 monoclonal anti-XRCC1 antibody (clone 33–2–5; Interchim, Montlucon, France). Negative control immunohistochemical experiments were systematically performed with the exclusive omission of each primary antibody. For immunostaining of 8-oxoG in DNA, ribonucleic acids were removed from sections with 20 µg/ml Rnase solution (Invitrogen) and tissue DNA was denatured in 2N HCl for 5 min. Sections were then incubated with the primary antibody and labeled using a detection kit (ChemMate; Dako) with biotinylated secondary antibody and diaminobenzidine (DAB) as the substrate. After DAB staining, tissue sections were counterstained with methyl green solution and mounted with Eukitt (PolyLabo).

### Determination of Ogg1 and AP-lyase activities in the neuroretina

Proteins were extracted from neuroretina by sonication (8×1 s pulses) in lysis buffer containing 250 mM NaCl, 20 mM Tris-HCl, 1 mM EDTA (pH 8) supplemented with protease inhibitors (0.8 µg/ml aprotinin, 0.8 µg/ml antipain, and 0.8 µg/ml leupeptin). This was followed by centrifugation (85000x g for 30 min at 4 °C). 8-oxoG DNA glycosylase assays were performed as previously described [20]. Briefly, 5 µg protein was incubated for 60 min with a 34-mer oligonucleotide (50 fmol) containing a [γ^32^P]-radiolabeled 8-oxoG:C duplex. Reactions were stopped by addition of NaOH to a final concentration of 0.1 M and incubation for 15 min at 37 °C to cleave the abasic sites. Products were resolved by denaturating 7M urea-20% PAGE and gels were scanned in a storm PhosphoImager.

For APE1 activity reactions, a 34 mer oligodeoxynucleotide containing a THF was labeled at the 5′ end using [γ-^32^P]ATP (3,000 Ci/mmol; Amersham) and T4 polynucleotide kinase (New England Biolabs, Saint Quentin Yvelines, France). The ^32^P-labeled strand was hybridized with a complementary sequence. In a standard reaction (16 µl of final volume), 50 fmol of the duplex oligonucleotide were incubated with 10 ng of the protein extract for 30 min at 37 °C in a buffer containing 25 mM Tris-HCl, 1 mM MgCl_2_ and 0.4 mg/ml BSA at a pH 8. Reactions were stopped with 6 µl of formamide dye and heated for 5 min at 95 °C. Products were resolved by denaturating 7M urea-20% PAGE and gels were scanned in a storm PhosphoImager.

### Statistical analysis

Statistical analysis was performed using ANOVA and standard Student two-tailed *t*-test (ANOVA, Statview software program, version 5) to detect significant intergroup differences. Values are means±SEM, and p<0.05 was considered statistically significant.

## Results

### Presence of 8-oxoG in the DNA of mouse retinal cells

**Figure 1:**
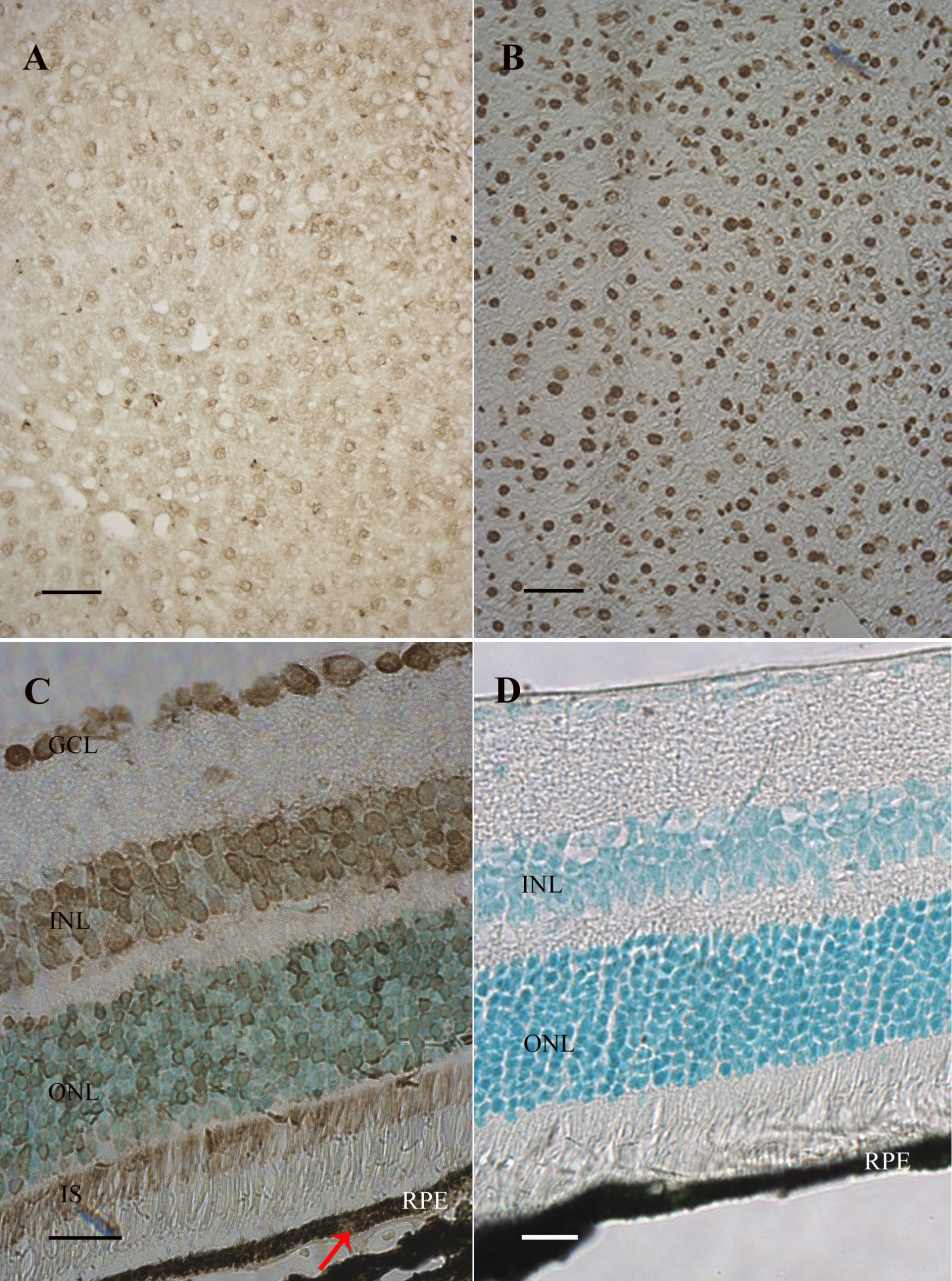
Immunohistological detection of 8-oxoguanine in the mouse retina. Immunostaining using an antibody raised against 8-oxoguanine (8-oxoG) was detected in liver of wild-type (**A**) and Ogg1-deficient mice (**B**), and in C57BL/6J mouse retina (**C**). A stronger signal was detected in liver of Ogg1-deficient mice as compared to wild-type mice. This confirmed the accumulation of the modified base. 8-oxoG was present in all retinal nuclear layers, in photoreceptor inner segments (IS), and in retinal pigment epithelium (RPE; arrow). The 8-oxoG labeling appears in brown. No signal was detected when the specific anti-8-oxoG antibody was omitted (**D**). Scale bar equals 50 µm in **A** and **B**, 25 μm in **C**, and 60 µm in **D**. Abbreviations: ganglion cell layer (GCL); inner nuclear layer (INL); outer nuclear layer (ONL).

The relative steady-state level of 8-oxoG in retinal cells was determined by immunohistochemistry using the anti-8-oxoG monoclonal antibody N45.1, which is highly specific. Indeed, its labeling specificity has been previously established in various tissues [21-24]. Liver, an organ with a low cell proliferation rate, was used as control. The presence of stable 8-oxoG residues in the genome of wild-type mouse liver and their increased frequency in the liver of Ogg1-deficient mice has been previously demonstrated and reported [14]. Using immunohistochemistry, we confirmed that 8-oxoG was formed in basal conditions in several hepatocytes (Figure 1A). We observed a stronger signal in Ogg1 null mice liver tissue sections (Figure 1B), confirming the accumulation of 8-oxoG in the liver of deficient mice and the specificity of the antibody. No 8-oxoG immunostaining was observed in the appropriate control experiments (Figure 1D). We demonstrated that 8-oxoG was abundant under basal conditions in the various retinal layers: ganglion cell layer (GCL), inner nuclear layer (INL), outer nuclear layer (ONL), photoreceptor inner segments (IS; Figure 1C). Nevertheless, our result showed that relative labeling differed from cell to cell in the inner and outer retinal cell layers. In addition, we also observed a specific immunolabeling in the retinal pigment epithelium (RPE; Figure 1C).

### *Ogg1* mRNA is produced in mouse retina

**Figure 2:**
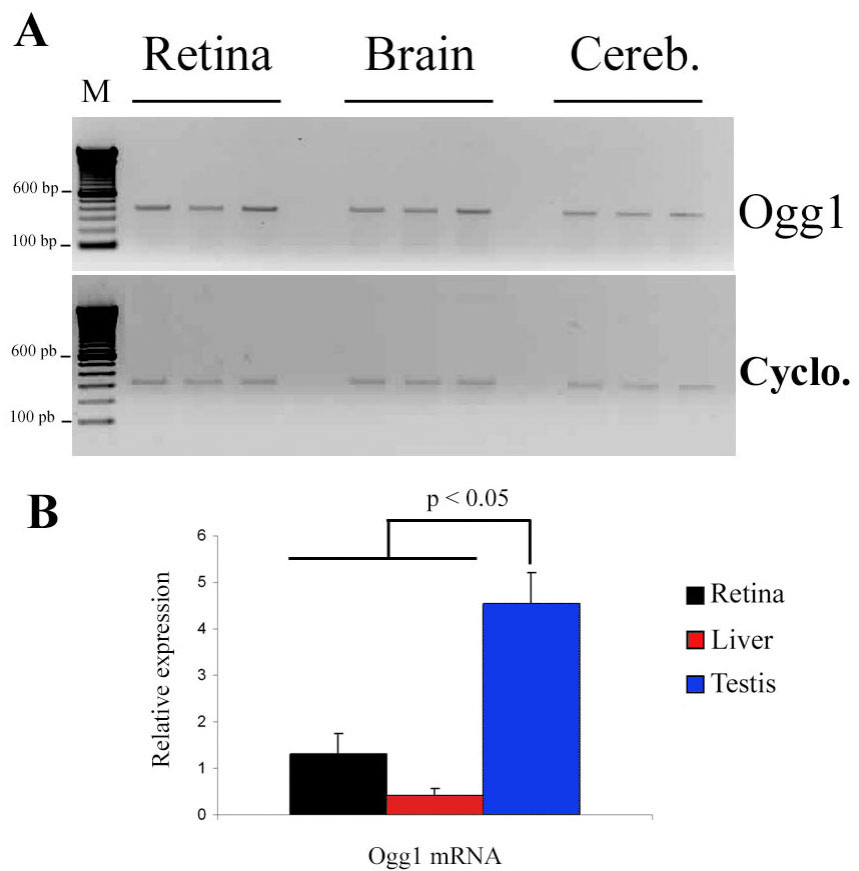
*Ogg1* messenger in mouse retina, forebrain and cerebellum with associated densitometric analysis of the PCR bands. **A** shows relative amount of 8-oxoguanine glycosylase (*Ogg1*) mRNA in the mouse retina, forebrain (brain) and cerebellum (Cereb). To determine the expression of *Ogg1* mRNA in adult C57BL/6J mouse neuroretinal cells (n=3), forebrain (n=3), and cereb (n=3), we performed semiquantitative RT–PCR using specific primers for mouse *Ogg1* mRNA and cyclophilin A mRNA (*Cyclo*). *Cyclo* mRNA was used as an internal control. The 400 bp band corresponds to *Ogg1* PCR products and the 311 bp band corresponds to cyclophilin A PCR products. **B** represents comparison of the *Ogg1* mRNA expression in neuroretina, liver, and testis. Quantitative RT–PCR was performed to determine the relative levels of *Ogg1* mRNA in adult C57BL/6 mouse neuroretina (n=5), liver (n=5), and testis (n=5), *Cyclo* mRNA was used as an internal standard for normalization. Significant, higher levels of *Ogg1* mRNA expression were observed in testis as compared to those obtained in the liver and the neuroretina. Values are means±SEM.

Using semiquantitative RT–PCR analyses, we determined the expression of *Ogg1* mRNA in the adult mouse neuroretina. (Figure 2A). The housekeeping gene, *cyclophilin* A, was used as a common internal standard (311 bp product). RT–PCR was also performed on mRNA extracted from mouse cerebellum and forebrain neuronal tissues in which *Ogg1* gene expression has previously been described [25]. RT–PCR with mouse neuroretina RNA yielded an expected 400 bp product similar to that amplified from cerebellum and forebrain, and it seemed that there was no difference in *Ogg1* mRNA levels between the three postmitotic tissues studied. Using quantitative RT–PCR analyses, we detected significantly higher level of *Ogg1* mRNA expression in testis than in the liver, as previously described [26,27], and the neuroretina (Figure 2B). In addition, we showed that the amounts of *Ogg1* mRNA were similar in neuroretina and liver (p=0.557).

**Figure 3:**
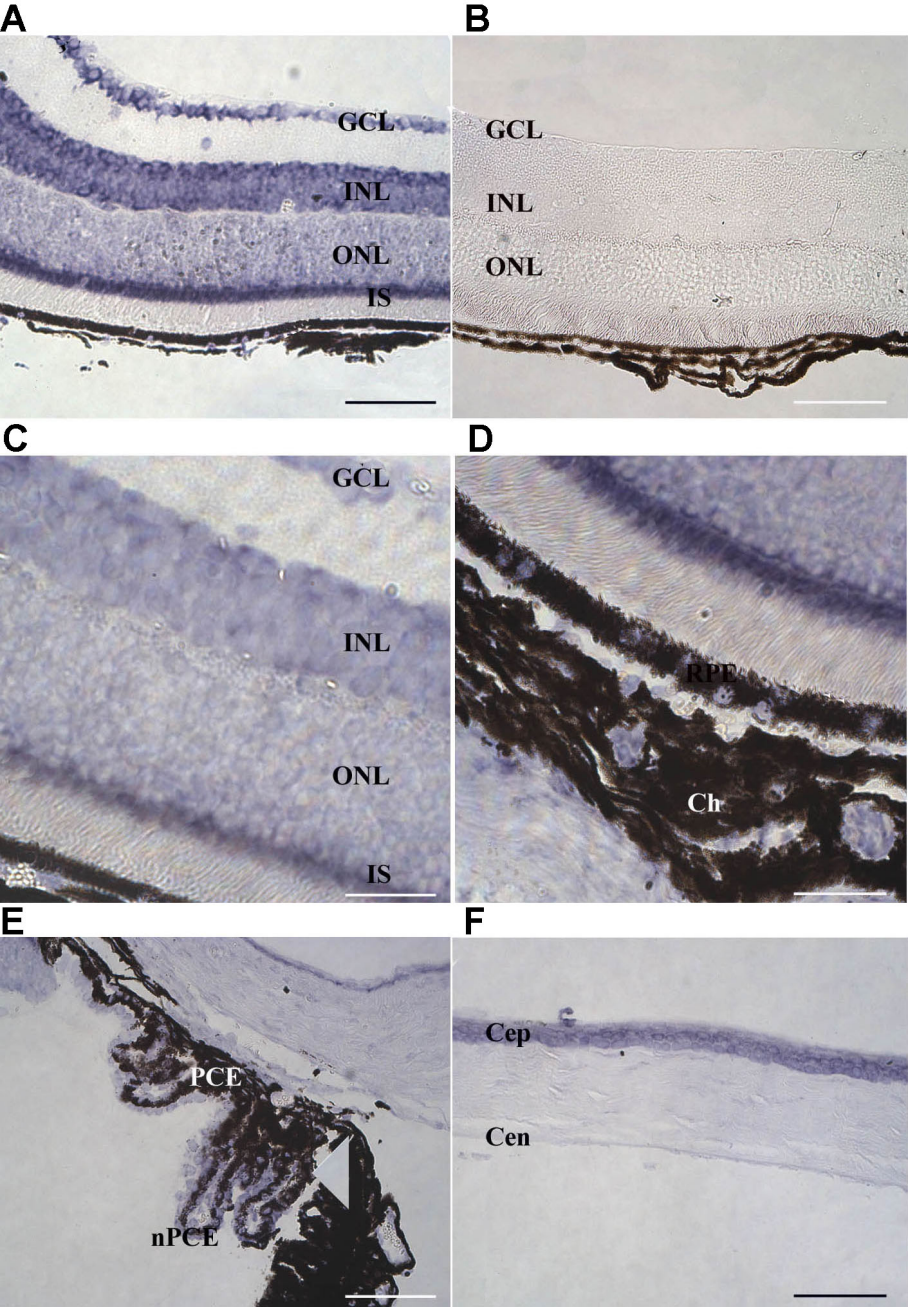
Distribution of *Ogg1* transcript in the mouse adult eye. In situ hybridization signals of 8-oxoguanine glycosylase (*Ogg1*) mRNA were detected in neuroretina **(A, C,** and **D**), ciliary body (**E**), and cornea (**F**) using a specific antisense digoxigenin-labeled riboprobe. Labeled *Ogg1* mRNA appears as purple precipitates. No signal was detected with a control sense probe (**B**). *Ogg1* mRNA was present in all retinal nuclear layers, in photoreceptor inner segments (IS), and in retinal pigment epithelium (RPE). *Ogg1* mRNA was also detected in non-neuronal cells: corneal epithelial (Cep) and endothelial cells (Cep), keratocytes of the corneal stroma, pigmentary epithelium (PCE), and nonpigmentary ciliary epithelium (NPCE). **C** and **D** represent a high magnification of the inner portion of the neuroretina, the retinal pigment epithelium (RPE), and the choroid (Ch), respectively. Scale bar equals 70 μm in **A, B, E**, and **F**, and 25 µm in **C** and **D**. Abbreviations: ganglion cell layer (GCL); inner nuclear layer (INL); outer nuclear layer (ONL).

We performed in situ hybridization on paraffin-embedded sections of adult C57BL/6 mouse eyes to determine the tissue distribution of *Ogg1* transcripts. No signal was observed in the retina using the *Ogg1* sense probe (Figure 3B). A strong *Ogg1* mRNA signal was detected in several cell layers of the neuroretina: GCL, INL, ONL, and IS (Figure 3A,C,D). No labeling was observed in the inner and outer plexiform layers. *Ogg1* mRNA was also present in RPE cells. These cells, like the neuroretina, are derived from the prosencephalon. *Ogg1* transcripts were also detected in other ocular cells derived from the nervous system such as choroidal cells (melanocytes derived from the neural crest, unlike RPE cells derived from the CNS), and ciliary body epithelial cells (pigmented and nonpigmented, Figure 3E). In non-neuronal cells, *Ogg1* transcripts were present in corneal epithelial (Cep) and endothelial cells (Cen) as well as in keratocytes of the corneal stroma (Figure 3F).

### Ogg1 protein is abundantly produced in mouse retina

**Figure 4:**
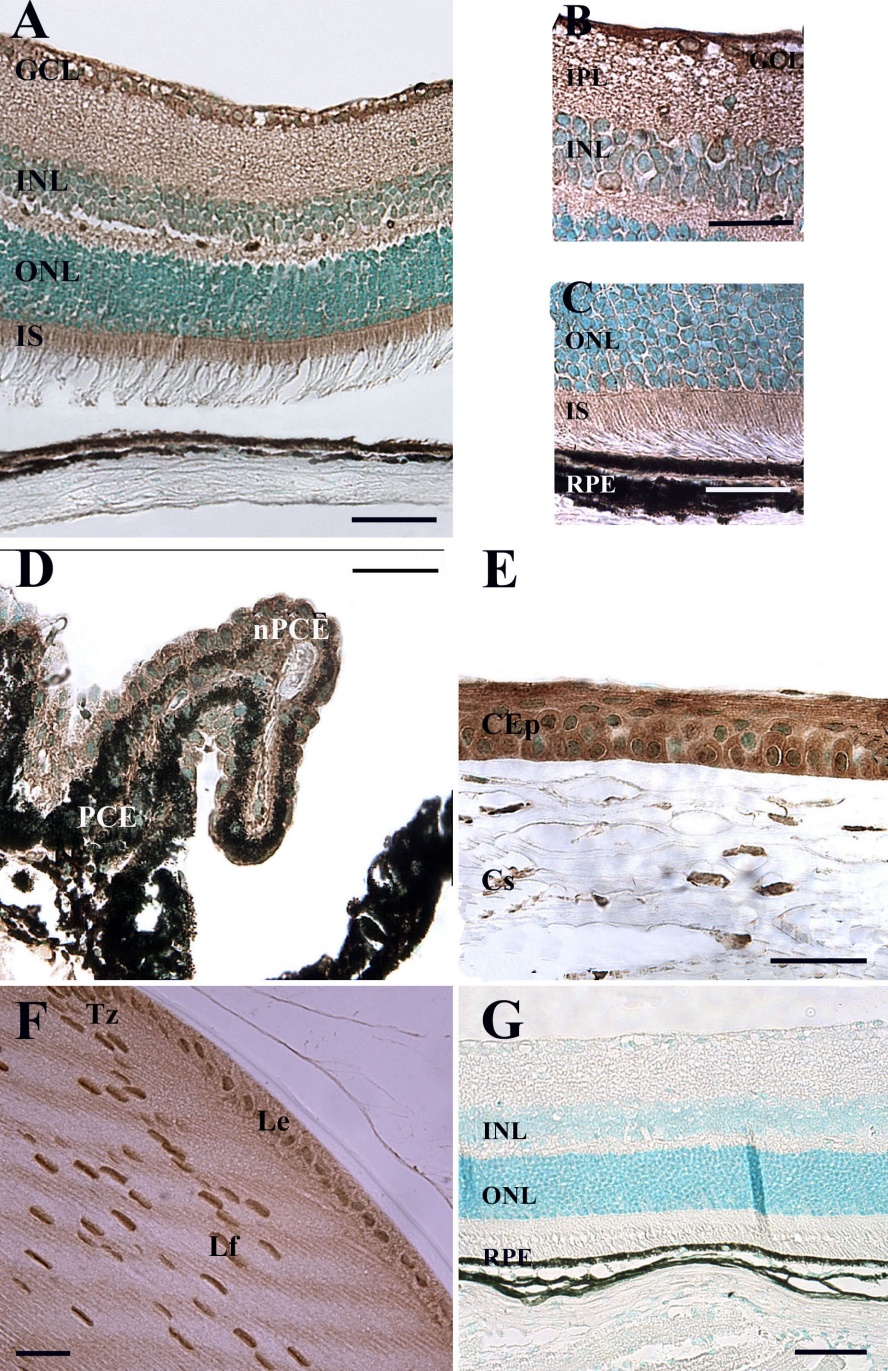
Immunohistochemical localization of Ogg1 in the adult mouse eye. Using an antibody raised against human 8-oxoguanine DNA glycosylase, immunostaining is detected in in neuroretina (**A**),ciliary body (**D**), cornea (**E**), and lens (**F**). The Ogg1 staining appears in brown, and sections were counterstained with methyl green solution. No signal was detected when the specific anti-Ogg1 antibody was omitted (**G**). Ogg1 protein was mainly present in the ganglion cell layer (GCL), in photoreceptor inner segments (IS), and in the outer (OPL) and inner plexiform layers (IPL). **B** and **C** show a high magnification of the inner and the outer portions of retina, respectively. Ogg1 protein was also detected in non-neuronal cells: corneal epithelium (Cep), pigmentary epithelium (PCE) and nonpigmentary ciliary epithelium (NPCE), lens epithelium (Le), lens transition zone (Tz), and lens fibers (Lf). Scale bar equals 50 μm in **A, D**,and **G**, 20 μm in **B, C,** and **E**, and 45 µm in **F**. Abbreviations: corneal stroma (Cs); inner nuclear layer (INL); outer nuclear layer (ONL); retinal pigment epithelium (RPE).

The presence of Ogg1 protein in the eye was studied by immunohistochemistry using a polyclonal antibody raised against human Ogg1. Its specificity has been confirmed in previous studies performed in various tissues by western blotting [28-31]. In addition, we did not detected any signal in sections treated without primary antibody (Figure 4G). The strongest Ogg1 immunolabeling in mouse retina was only detected in the GCL and the IS (Figure 4A-C). We also observed Ogg1 immunolabeling in the two retina plexiform layers (OPL and IPL) containing nerve cell processes and synapses (Figure 4A-C). As was the case for *Ogg1* mRNA (Figure 3), Ogg1 protein was present in non-neuronal tissues: the corneal epithelial cells (Figure 4E), the lens equatorial epithelial cells of the germinative zone, the lens anterior epithelium, and early differentiating lens fiber cells (Figure 4F).

**Figure 5:**
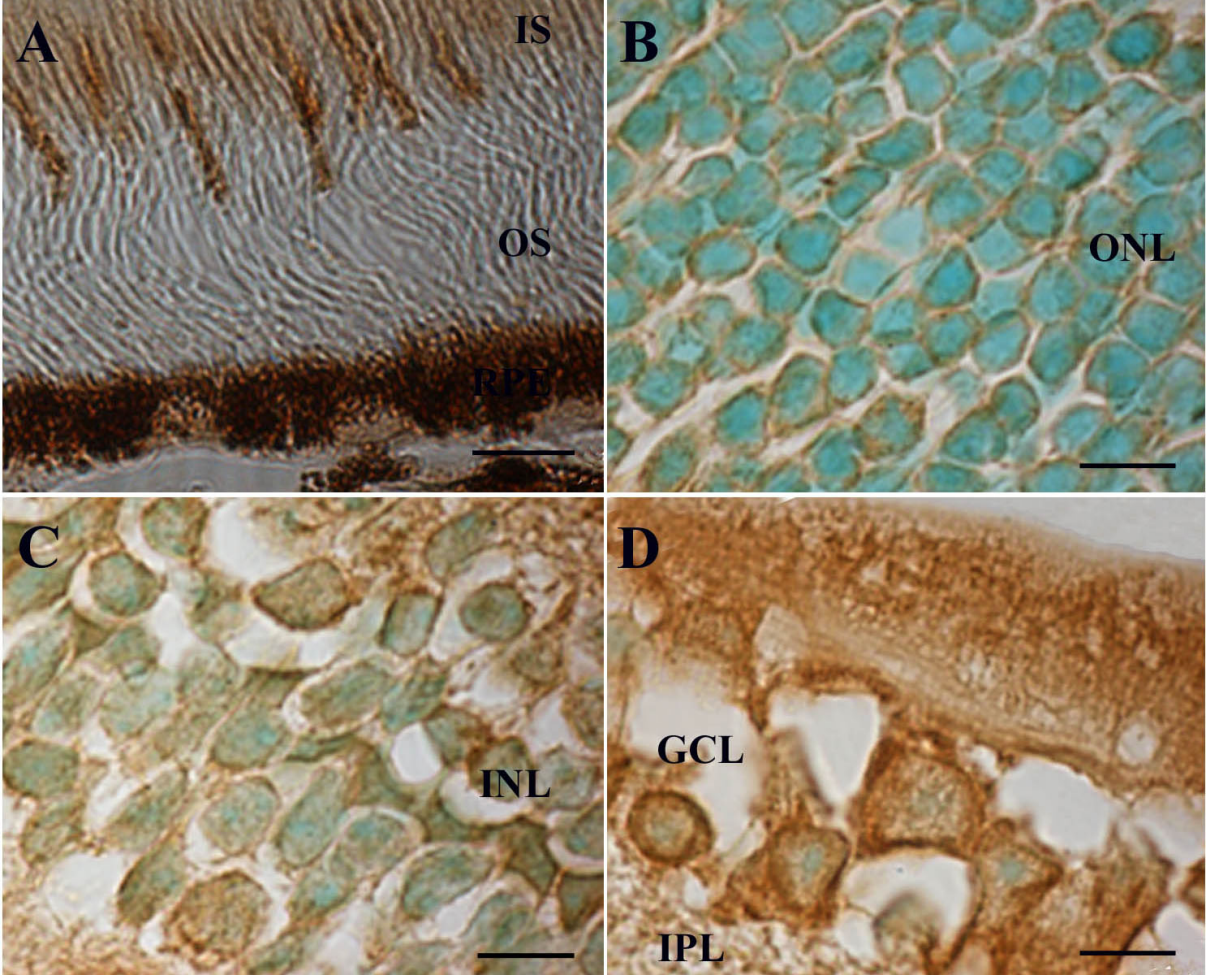
Cellular distribution of Ogg1 protein in the retinal cells. Using an antibody raised against human 8-oxoguanine DNA glycosylase, immunostaining is detected in photoreceptor inner segments (**A**), outer nuclear layer (ONL, **B**), inner nuclear layer (INL, **C**) and ganglion cell layer (GCL, **D**). Ogg1 immunoreactivity was detected both in the cytoplasmic and nuclear compartments of the immunolabeled cells of GCL and INL. The staining seemed only cytoplasmic in photoreceptor cells, which also displayed a strong labeling in the inner segments (IS). Scale bar equals 20 μm in **A**, and 8 µm in **B, C,** and **D**. Abbreviations:, inner plexiform layer (IPL); outer segment (OS); outer plexiform layer (OPL); retinal pigment epithelium (RPE).

Examination of higher magnification images revealed that the INL, the ONL, and the RPE were also stained (Figure 4B,C and Figure 5A-C). These images allowed us to determine the intracellular localization of Ogg1 protein in retinal cells. In photoreceptor cells, Ogg1 labeling was concentrated only in the IS and cytoplasm (mainly around the nuclear periphery; Figure 5A,B). No signal was detected in photoreceptor cell nuclei. By contrast, Ogg1 protein was mainly present in the cytoplasm in INL and ganglion cells (Figure 5C,D) but we observed some Ogg1 immunoreactivity localized in the nuclei of those cells. Identical intracellular localizations of Ogg1 protein in neuroretina were observed with sections not counterstained with a green methyl solution (data not shown). These data confirmed the results of the in situ hybridization analysis and demonstrated that high Ogg1 levels are produced in neural and non-neural cells of the mouse eye.

### Other BER proteins are present in the mouse retina

**Figure 6:**
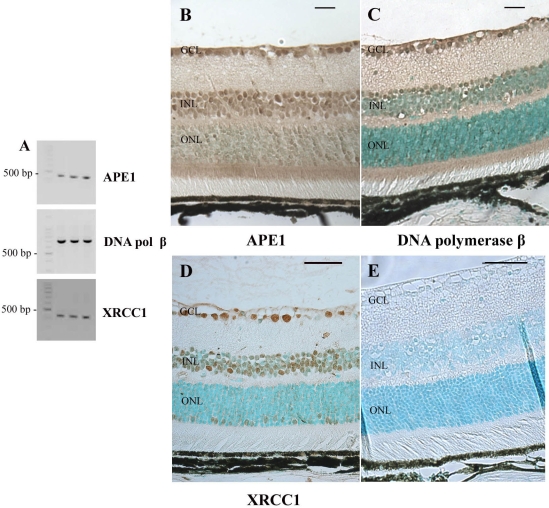
Expression of BER mRNA and proteins in the adult mouse retina. **A**: Semiquantitative RT–PCR experiments were performed to determine the *APE1*, DNA polymerase β (*DNA pol β*), and *XRCC1* mRNA levels of expression in C57BL/6 mouse neuroretinal cells. Cyclophilin A (*Cyclo*) was used as an internal control. Specific primers for amplifying mouse *APE1, DNA pol β, XRCC1,* and Cyclophilin A cDNAs were used. The expected size for each specific amplified product was obtained: 446 bp for *APE1*, 693 bp for *DNA pol β*, 418 bp for *XRCC1*, 311 bp for *Cyclophilin A*. Immunohistological localization of APE1, DNA polymerase β, and XRCC1 in the adult mouse retina was performed using an antibody raised against APE1 (**B**), DNA pol β (**C**) or XRCC1 (**D**). The staining appears in brown. Sections were counterstained with a methyl green solution. No signal was detected when the specific anti-APE1 antibody was omitted (**E**). APE1 and DNA polymerase β were detected in the ganglion cell layer (GCL), the inner nuclear layer (INL), and the photoreceptor inner segments (IS). Labeling was also observed in the INL and outer plexiform layers (ONL). Surprisingly, XRCC1 was not detected in the IS. Scale bar equals 50 μm in **B, C,** and **E**, and 10 µm in D.

When initiated by Ogg1, the base excision repair of 8-oxoG involves a highly coordinated process mediated by several proteins [20]. Using both RT–PCR and immunohistochemistry, we determined whether APE1, DNA polymerase β, and XRCC1, all of which are involved in BER, were present in mouse neuroretina. Amplification of *APE1* (446 bp), *DNA polymerase β* (693 bp), and *XRCC1* (418 bp), through the use of specific primers, confirmed the expression of these genes in the mouse retina (Figure 6A).

Using an antibody raised against human APE1, we detected this protein in GCL, INL, IS, IPL, and OPL (Figure 6B). Weaker staining was also present in the ONL. Similarly, DNA polymerase β was present in the GCL, the inner nuclear cell layer (mostly localized in the deep inner part of this layer), the IS, and the plexiform layers (Figure 6C). Surprisingly, light staining was found for XRCC1 (Figure 6D). Indeed, this protein was only detected in ganglion and INL. Some cells seemed to show immunostaining in the photoreceptor cell nuclear layer, but we did not detect any staining in the IS. Table 2 summarizes the results obtained in the immunohistochemistry experiments with the anti-8-oxoG, -Ogg1, -APE1, DNA polymerase β, and -XRCC1 antibodies and presented in Figure 1, Figure 4, Figure 5, and Figure 6.

**Table 2:** Expression of 8-oxoG and BER in the adult mouse ocular tissues.

	**Retina**							**Non-neuronal tissues**		
	**GCL**	**INL**	**ONL**	**IS**	**IPL**	**OPL**	**RPE**	**Cep**	**Le**	**Lf**
*8-oxoG*	+	+	+	+	-	-	+	ns	ns	ns
*Ogg1*	+ + +	+	+	+ + +	+	+	+	+	+	+
	nuclear and cytoplasmic	nuclear and cytoplasmic	cytoplasmic							
*APE1*	+ +	+ +	+	+ +	+	+	+	ns	ns	ns
*DNA pol β*	+ +	+ +	+	+ +	+	+	-	ns	ns	ns
*XRCC1*	+ +	+ +	+	-	-	-	+	ns	ns	ns

The cellular localizations of 8-oxoG, Ogg1, APE1, DNA pol β, and XRCC1 observed by immunohistochemistry using the appropriate specific antibodies are present in Table 2. The table summarizes the results presented in Figure 1, Figure 4, Figure 5, and Figure 6. Plus (+) means that one of these modified base or enzymes: 8-oxoG, Ogg1, APE1, DNA pol β, and XRCC1 is expressed in a specific retinal layer or sublayer and, whenever it was possible to determine it unequivocally, in a specific cellular compartment of a retinal cell type: nucleus and/or cytoplasm. Minus (-) means that one of these modified base or enzymes: 8-oxoG, Ogg1, APE1, DNA pol β, and XRCC1 is not expressed and ns means not shown.

### 8-oxoG DNA glycosylase and AP-endonuclease activity detected in mouse neuroretina

**Figure 7:**
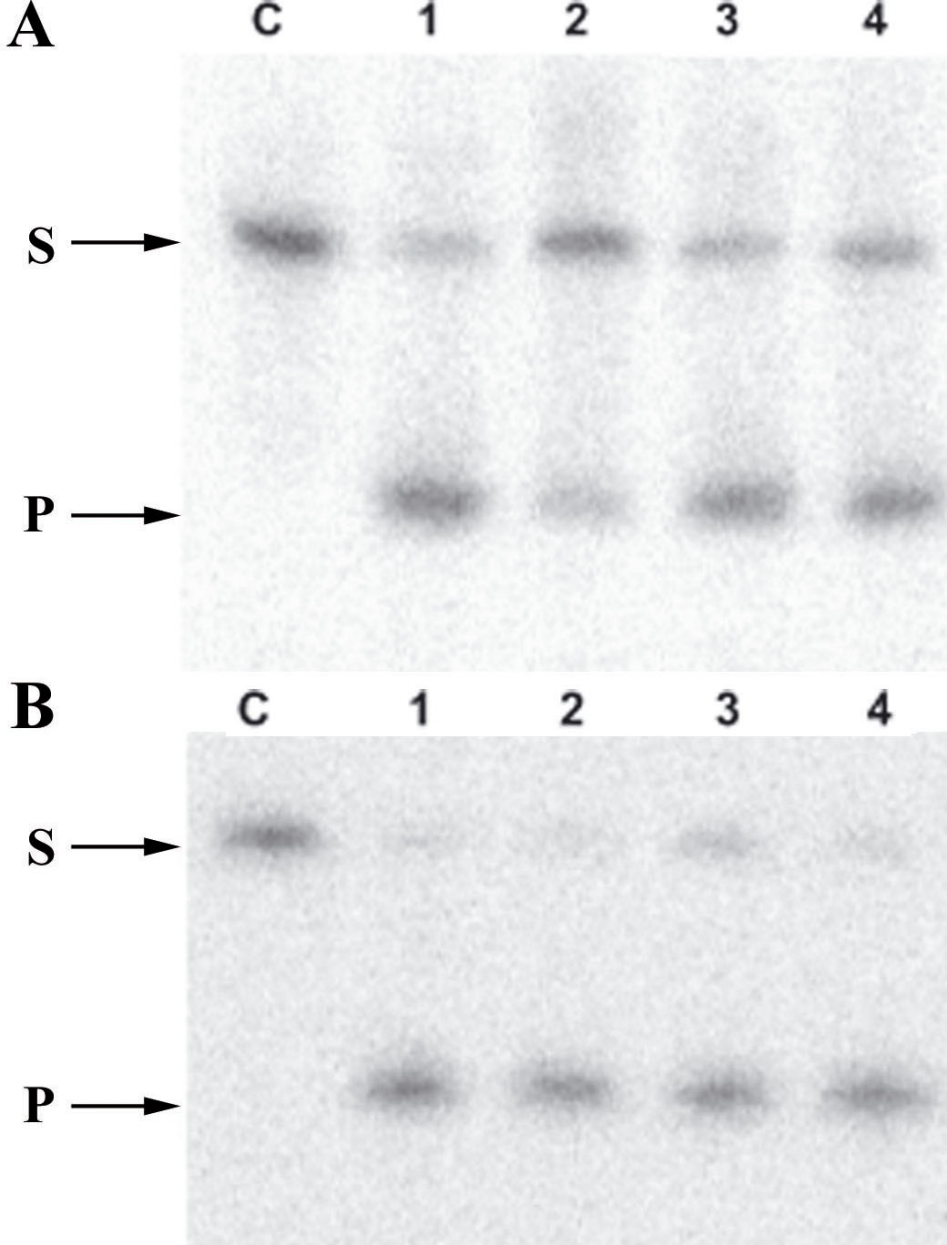
BER activities in neuroretina protein extracts. Representative gels showing the cleavage of the double-stranded radiolabeled oligonucleotide substrate containing 8-oxoG: C (**A**) and an abasic site analog (**B**). Lane C corresponds to experiments omitting protein extract used as negative control. Lanes 1 to 4 correspond to enzymatic activities detected in protein extracts from the adult mouse neuroretina. (S) indicates the substrate DNA and (P) the cleaved product.

We determined whether the mRNA and protein expression detected were associated with enzymatic activity in the retinal tissues. This we did by testing the capacity of cell extracts to excise an 8-oxoG or cleave an AP site analog through the use of double-stranded oligonucleotides harboring either an 8-oxoG residue or a tetrahydrofuran. Four independent cell extracts obtained from adult mouse neuroretina showed 8-oxoG-specific DNA glycosylase activity, as revealed by the detection of the cleaved oligonucleotide (Figure 7A). Similarly, when the same extracts were incubated in the presence of a DNA substrate carrying an AP site analog, they were able to efficiently cleave the DNA at the lesion (Figure 7B). These results demonstrated that the neuroretina is proficient for the initial steps of the BER of 8-oxoG and abasic sites.

## Discussion

In this study, we found that Ogg1 is abundant in ganglion cells, in the INL, including amacrine, bipolar, horizontal cells, and Müller cell nuclei, as well as in the inner segments of photoreceptor cells. The adult mouse retinal Ogg1 distribution observed is consistent with the sites of production of 8-oxoG. These results support the recent data of Wang et al. [31,32]. Most important, our study is the first to demonstrate the expression and cellular localization of APE1, DNA polymerase β, and XRCC1 in the mouse retina and thus provides further evidence to support the existence of a functional BER pathway in this tissue.

Our findings suggest that 8-oxoG in retinal neuronal DNA is likely to be recognized by Ogg1. Indeed, we demonstrated that high levels of *Ogg1* mRNA and protein are detected in all neuronal cell populations and RPE cells. This is consistent with the distribution of this glycosylase in the mouse CNS [25,33] and might reflect a link between Ogg1 and maintenance of cellular integrity in neuronal cells. The abundant production of this DNA glycosylase highlights the importance of recognition of 8-oxoG in postmitotic tissues in the eye, in particular neuroretina and RPE, both characterized by a high level of cellular activity. Indeed, photoreceptor cells continuously produce high levels of ROS, which are byproducts of the various functions of these cells throughout their lifespan. RPE cells are critical for the maintenance of retinal homeostasis; they are involved in regeneration of the visual pigments, regulation of visual transduction, transport of retinoids and nutrients to the photoreceptor cells, and phagocytosis of the oldest synthesized photoreceptor outer segments (POS; for review, see [34]). The role of RPE cells in the absorption of stray light further exposes these cells to additional oxidative stress induced by light. As recently described, our study confirms both the recently reported presence of 8-oxoG and the expression of Ogg1 in RPE cells [31].

In neurodegenerative disorders affecting the brain and retina, mitochondrial DNA damage induced by oxidative stress is a major factor leading to neuronal dysfunction and death. The mitochondrial electron transport chain is a primary source of ROS in cells and, in the brain, 8-oxoG production is 10 to 15 times higher in mitochondrial DNA than in nuclear DNA [35-38]. The role of *Ogg1* gene product in the prevention of 8-oxoG was demonstrated by the substantial accumulation of oxidized guanine in the mitochondrial DNA in Ogg1-deficient mice [39]. In this study, we showed that Ogg1 is abundant in IS, well known to be major sites of mitochondria localization in photoreceptor cells. We also detected Ogg1 in the plexiform layers, where it might correspond to a pool of Ogg1 protein transported to neuronal axon terminals. These neuronal structures have also a rich content of mitochondria. Our results highlight the involvement of Ogg1 in mitochondrial DNA repair in mouse retinal cells. This is consistent with previous findings showing the accumulation of 8-oxoG in IS and synaptic terminals and subsequent upregulation of Ogg1 6 h after induction of oxidative stress by bright light [40]. In RPE cells, Wang et al. [31] also described greater levels of 8-oxoG in mitochondrial DNA than in nuclear DNA. In addition, the increase of mitochondrial damage in RRE cells from old rodents seemed related to a decrease of DNA repair capabilities. This correlation suggests a potential link between repair of oxidative DNA damage and the development of age-related macular degeneration. This observation emphasizes the importance of 8-oxoG repair in retinal mitochondria. In spite of risks of DNA damage due to high oxygen consumption and high metabolic rates, no degenerative processes in brain and neuroretina have been described so far in Ogg1-deficient mice. The absence of degenerative processes might be explained by the involvement of “back up” repair pathways of 8-oxoG such as nucleotide excision repair or transcription coupled repair, as previously described [41]. These systems may be sufficient to maintain the integrity of the DNA molecule and prevent cellular dysfunction and apoptosis. Nevertheless, as we observed in retinal neurons, these “backup” repair pathways might not be efficient enough to keep the steady levels of 8-oxoG sufficiently low for allowing normal retinal functions. Moreover, It has been described that several DNA glycosylases are able to recognize and excise the 8-oxoG [42]. The combination of their enzymatic activities likely contribute to prevent the accumulation of this oxidative DNA lesion.

In addition, we also found in this study that Ogg1 immunostaining is primarily cytoplasmic. In humans, alternative splicing was shown to determine differential intracellular localization of Ogg1 [43]. Using mouse embryo fibroblasts, it has been demonstrated that Ogg1 is mainly detected in cytoplasm with a diffuse distribution in nucleus. This is in agreement with our results [44]. Furthermore, it is generally admitted that most Ogg1 isoforms are cytoplasmic [43,45-48]. The repair of 8-oxoG in retinal cell nuclear DNA may depend on redistribution of cytoplasmic Ogg1 to the nucleus, as previously observed in mouse fibroblasts submitted to oxidative stress [44].

This study demonstrates the unequivocal detection of Ogg1 enzymatic activity as well as that of the AP endonuclease APE1, in extracts from adult mouse retinal cells. The detection of Ogg1 and APE1 enzymatic activities, the two first enzymes of the base excision repair pathway, in the adult mouse retina strongly suggests that the whole BER pathway is fully functioning in retinal cells during physiologic conditions of retina activities. We then used immunohistochemistry to demonstrate the presence of the proteins APE1, DNA polymerase β, and XRCC1 that are involved in the BER steps following the excision of the 8-oxoG in retinal cells. Furthermore, the cellular distribution of these proteins was similar to that of Ogg1. These findings show that retinal cells express a complete BER pathway. It is likely that these proteins interact with each other, as has been previously demonstrated in other tissues, including the brain (for reviews, see [13,49,50]). Formation of such complexes may increase the specificity and efficiency of the BER pathway. In particular, APE1 has been shown to enhance glycosylase activity and displace the glycosylase from the 8-oxoG lesion [51-54]. However, XRCC1 was only detected in ganglion cells, in INL cells and in outer nuclear cells of the ONL. No significant XRCC1 immunoreactivity was found within IS where the levels of Ogg1, APE1, and DNA polymerase β are high. XRCC1 is a scaffold protein, interacting with many other BER proteins including Ogg1, APE1, DNA polymerase β. and Ligase IIIα [13,20,52,55-57]. XRCC1 is recruited to damaged sites to coordinate the interaction of BER proteins and regulate their activities (for review, see [49]). Both the embryonic lethality of XRCC1-deficient mice and the genetic instability of cells lacking this protein demonstrate the critical role of XRCC1 [58]. The absence of XRCC1 in IS, which contrasts strikingly with its presence in both inner nuclear and ganglion cells layers, suggests that the elimination of the 8-oxoG in the retina occurs and is coordinated through two pathways that differ slightly according to the cellular localization of 8-oxoG. XRCC1, although clearly involved in BER, is an almost exclusively nuclear protein. Thus, mitochondrial BER might be able to process without it. Indeed, the repair of 8-oxoG can be reconstituted in vitro in the absence of XRCC1 [59]. Of utmost importance is the fact that mitochondria of photoreceptor cells are mostly confined to inner segments. Our findings suggest that the pathway leading to 8-oxoG elimination in mitochondrial and nuclear DNA might differ.

In conclusion, we determined the cellular localization and activity of the proteins involved in the initial step of the base excision repair of 8-oxoG in the mouse retina. This study is the first to demonstrate the presence of a functional 8-oxoG BER system in retinal neurons. Further studies are required for unraveling the mechanisms of oxidative DNA damage repair in the mitochondria and nucleus of photoreceptor cells as well as in all other retinal neurons. Although there is not yet any unequivocal direct mechanistic link demonstrated between the accumulation of 8-oxoG, mitochondrial dysfunctions, oxidative stress, and ophthalmic diseases, several lines of evidences have been obtained recently that strongly support the existence of such links in AMD, glaucoma, and inherited retinal dystrophies [60-62]. Despite the fact that aging is characterized by an increasing load of mitochondrial and nuclear somatic mutations, albeit at a lower level in retinal cell nuclei, adult and aging retina appear to be resistant to the occurrence of cancers and seem to be endowed by strong mechanisms for eliminating sources of somatic mutations and especially efficient DNA repair systems. Aging and neurodegenerative diseases share frequently overlapping mechanisms. Age-related ophthalmic diseases such as age macular degenerations, many forms of glaucoma and cataracts are often associated with other neurodegenerative diseases. Additional experiments still remained to be performed for establishing accurately the possible roles of the accumulation of 8-oxoG, as well as other DNA lesions, and decreased DNA repair capabilities linked to aging in the pathogenesis of ophthalmic, often, age-related diseases.

## References

[1] Menu dit Huart L, Lorentz O, Goureau O, Léveillard T, Sahel JA (2004). DNA repair in the degenerating mouse retina.. Mol Cell Neurosci.

[2] Weissman L, de Souza-Pinto NC, Stevnsner T, Bohr VA (2007). DNA repair, mitochondria, and neurodegeneration.. Neuroscience.

[3] Halliwell B, Gutteridge JMC. Free Radicals in Biology and Medicine. 3rd ed. Oxford: Clarendon Press; 1999.

[4] Yu DY, Cringle SJ (2006). Oxygen distribution in the mouse retina.. Invest Ophthalmol Vis Sci.

[5] Cringle SJ, Yu PK, Su EN, Yu DY (2006). Oxygen distribution and consumption in the developing rat retina.. Invest Ophthalmol Vis Sci.

[6] Sickel W. Retinal metabolism in dark and light. In: Dartnall HJ, editor. Handbook of Sensory Physiology. Vol. VII. Berlin: Springer-Verlag; 1972. p. 662–727.

[7] Bazan NG (1989). The metabolism of omega-3 polyunsaturated fatty acids in the eye: the possible role of docosahexaenoic acid and docosanoids in retinal physiology and ocular pathology.. Prog Clin Biol Res.

[8] Mellerio J. Light effect on the retina. In: Albert DM, Jakobiec FA, editors. Principles and practice of ophthalmology: basic sciences. Philadelphia: WB Saunders; 1994. p. 1326–1345.

[9] Miceli MV, Liles MR, Newsome DA (1994). Evaluation of oxidative processes in human pigment epithelial cells associated with retinal outer segment phagocytosis.. Exp Cell Res.

[10] Tate DJ, Miceli MV, Newsome DA (1995). Phagocytosis and H2O2 induce catalase and metallothionein gene expression in human retinal pigment epithelial cells.. Invest Ophthalmol Vis Sci.

[11] Barzilai A, Biton S, Shiloh Y (2008). The role of the DNA damage response in neuronal development, organization and maintenance.. DNA Repair (Amst).

[12] Demple B, Harrison L (1994). Repair of oxidative damage to DNA: enzymology and biology.. Annu Rev Biochem.

[13] Campalans A, Marsin S, Nakabeppu Y, O'Connor TR, Boiteux S, Radicella JP (2005). XRCC1 interactions with multiple DNA glycosylases: a model for its recruitment to base excision repair.. DNA Repair (Amst).

[14] Klungland A, Rosewell I, Hollenbach S, Larsen E, Daly G, Epe B, Seeberg E, Lindahl T, Barnes DE (1999). Accumulation of premutagenic DNA lesions in mice defective in removal of oxidative base damage.. Proc Natl Acad Sci USA.

[15] Mamo S, Gal AB, Bodo S, Dinnyes A (2007). Quantitative evaluation and selection of reference genes in mouse oocytes and embryos cultured in vivo and in vitro.. BMC Dev Biol.

[16] Vandesompele J, De Preter K, Pattyn F, Poppe B, Van Roy N, De Paepe A, Speleman F (2002). Accurate normalization of real-time quantitative RT-PCR data by geometric averaging of multiple internal control genes.. Genome Biol.

[17] Suzuki T, Akimoto M, Mandai M, Takahashi M, Yoshimura N (2005). A new PCR-based approach for the preparation of RNA probe.. J Biochem Biophys Methods.

[18] Young ID, Stewart RJ, Ailles L, Mackie A, Gore J (1993). Synthesis of digoxigenin-labeled cRNA probes for nonisotopic in situ hybridization using reverse transcription polymerase chain reaction.. Biotech Histochem.

[19] Pequignot MO, Provost AC, Salle S, Taupin P, Sainton KM, Marchant D, Martinou JC, Ameisen JC, Jais JP, Abitbol M (2003). Major role of BAX in apoptosis during retinal development and in establishment of a functional postnatal retina.. Dev Dyn.

[20] Marsin S, Vidal AE, Sossou M, Menissier-de Murcia J, Le Page F, Boiteux S, de Murcia G, Radicella JP (2003). Role of XRCC1 in the coordination and stimulation of oxidative DNA damage repair initiated by the DNA glycosylase hOGG1.. J Biol Chem.

[21] Toyokuni S, Tanaka T, Hattori Y, Nishiyama Y, Yoshida A, Uchida K, Hiai H, Ochi H, Osawa T (1997). Quantitative immunohistochemical determination of 8-hydroxy-2'-deoxyguanosine by a monoclonal antibody N45.1: its application to ferric nitrilotriacetate-induced renal carcinogenesis model.. Lab Invest.

[22] Nakae Y, Stoward PJ, Bespalov IA, Melamede RJ, Wallace SS (2005). A new technique for the quantitative assessment of 8-oxoguanine in nuclear DNA as a marker of oxidative stress. Application to dystrophin-deficient DMD skeletal muscles.. Histochem Cell Biol.

[23] Kunisada M, Sakumi K, Tominaga Y, Budiyanto A, Ueda M, Ichihashi M, Nakabeppu Y, Nishigori C (2005). 8-Oxoguanine formation induced by chronic UVB exposure makes Ogg1 knockout mice susceptible to skin carcinogenesis.. Cancer Res.

[24] Ichiseki T, Kaneuji A, Katsuda S, Ueda Y, Sugimori T, Matsumoto T (2005). DNA oxidation injury in bone early after steroid administration is involved in the pathogenesis of steroid-induced osteonecrosis.. Rheumatology (Oxford).

[25] Verjat T, Dhenaut A, Radicella JP, Araneda S (2000). Detection of 8-oxoG DNA glycosylase activity and OGG1 transcripts in the rat CNS.. Mutat Res.

[26] Wellejus A, Bornholdt J, Vogel UB, Risom L, Wiger R, Loft S (2004). Cell-specific oxidative DNA damage induced by estrogen in rat testicular cells in vitro.. Toxicol Lett.

[27] Rosenquist TA, Zharkov DO, Grollman AP (1997). Cloning and characterization of a mammalian 8-oxoguanine DNA glycosylase.. Proc Natl Acad Sci USA.

[28] Chatterjee A, Mambo E, Zhang Y, Deweese T, Sidransky D (2006). Targeting of mutant hogg1 in mammalian mitochondria and nucleus: effect on cellular survival upon oxidative stress.. BMC Cancer.

[29] Hildrestrand GA, Diep DB, Kunke D, Bolstad N, Bjoras M, Krauss S, Luna L (2007). The capacity to remove 8-oxoG is enhanced in newborn neural stem/progenitor cells and decreases in juvenile mice and upon cell differentiation.. DNA Repair (Amst).

[30] Rachek LI, Grishko VI, Musiyenko SI, Kelley MR, LeDoux SP, Wilson GL (2002). Conditional targeting of the DNA repair enzyme hOGG1 into mitochondria.. J Biol Chem.

[31] Wang AL, Lukas TJ, Yuan M, Neufeld AH (2008). Increased mitochondrial DNA damage and down-regulation of DNA repair enzymes in aged rodent retinal pigment epithelium and choroid.. Mol Vis.

[32] Wang AL, Lukas TJ, Yuan M, Neufeld AH (2008). Age-related increase in mitochondrial DNA damage and loss of DNA repair capacity in the neural retina.. Neurobiol Aging.

[33] Araneda S, Mermet N, Verjat T, Angulo JF, Radicella JP (2001). Expression of Kin17 and 8-OxoG DNA glycosylase in cells of rodent and quail central nervous system.. Brain Res Bull.

[34] Strauss O (2005). The retinal pigment epithelium in visual function.. Physiol Rev.

[35] Yakes FM, Van Houten B (1997). Mitochondrial DNA damage is more extensive and persists longer than nuclear DNA damage in human cells following oxidative stress.. Proc Natl Acad Sci USA.

[36] Santos JH, Hunakova L, Chen Y, Bortner C, Van Houten B (2003). Cell sorting experiments link persistent mitochondrial DNA damage with loss of mitochondrial membrane potential and apoptotic cell death.. J Biol Chem.

[37] Mecocci P, MacGarvey U, Kaufman AE, Koontz D, Shoffner JM, Wallace DC, Beal MF (1993). Oxidative damage to mitochondrial DNA shows marked age-dependent increases in human brain.. Ann Neurol.

[38] Hayakawa M, Hattori K, Sugiyama S, Ozawa T (1992). Age-associated oxygen damage and mutations in mitochondrial DNA in human hearts.. Biochem Biophys Res Commun.

[39] de Souza-Pinto NC, Eide L, Hogue BA, Thybo T, Stevnsner T, Seeberg E, Klungland A, Bohr VA (2001). Repair of 8-oxodeoxyguanosine lesions in mitochondrial dna depends on the oxoguanine dna glycosylase (OGG1) gene and 8-oxoguanine accumulates in the mitochondrial dna of OGG1-defective mice.. Cancer Res.

[40] Cortina MS, Gordon WC, Lukiw WJ, Bazan NG (2005). Oxidative stress-induced retinal damage up-regulates DNA polymerase gamma and 8-oxoguanine-DNA-glycosylase in photoreceptor synaptic mitochondria.. Exp Eye Res.

[41] Le Page F, Klungland A, Barnes DE, Sarasin A, Boiteux S (2000). Transcription coupled repair of 8-oxoguanine in murine cells: the ogg1 protein is required for repair in nontranscribed sequences but not in transcribed sequences.. Proc Natl Acad Sci USA.

[42] Klungland A, Bjelland S (2007). Oxidative damage to purines in DNA: role of mammalian Ogg1.. DNA Repair (Amst).

[43] Nishioka K, Ohtsubo T, Oda H, Fujiwara T, Kang D, Sugimachi K, Nakabeppu Y (1999). Expression and differential intracellular localization of two major forms of human 8-oxoguanine DNA glycosylase encoded by alternatively spliced OGG1 mRNAs.. Mol Biol Cell.

[44] Conlon KA, Zharkov DO, Berrios M (2003). Immunofluorescent localization of the murine 8-oxoguanine DNA glycosylase (mOGG1) in cells growing under normal and nutrient deprivation conditions.. DNA Repair (Amst).

[45] Takao M, Aburatani H, Kobayashi K, Yasui A (1998). Mitochondrial targeting of human DNA glycosylases for repair of oxidative DNA damage.. Nucleic Acids Res.

[46] Shinmura K, Kohno T, Takeuchi-Sasaki M, Maeda M, Segawa T, Kamo T, Sugimura H, Yokota J (2000). Expression of the OGG1-type 1a (nuclear form) protein in cancerous and non-cancerous human cells.. Int J Oncol.

[47] Nakabeppu Y (2001). Regulation of intracellular localization of human MTH1, OGG1, and MYH proteins for repair of oxidative DNA damage.. Prog Nucleic Acid Res Mol Biol.

[48] Aburatani H, Hippo Y, Ishida T, Takashima R, Matsuba C, Kodama T, Takao M, Yasui A, Yamamoto K, Asano M (1997). Cloning and characterization of mammalian 8-hydroxyguanine-specific DNA glycosylase/apurinic, apyrimidinic lyase, a functional mutM homologue.. Cancer Res.

[49] Almeida KH, Sobol RW (2007). A unified view of base excision repair: lesion-dependent protein complexes regulated by post-translational modification.. DNA Repair (Amst).

[50] Izumi T, Wiederhold LR, Roy G, Roy R, Jaiswal A, Bhakat KK, Mitra S, Hazra TK (2003). Mammalian DNA base excision repair proteins: their interactions and role in repair of oxidative DNA damage.. Toxicology.

[51] Saitoh T, Shinmura K, Yamaguchi S, Tani M, Seki S, Murakami H, Nojima Y, Yokota J (2001). Enhancement of OGG1 protein AP lyase activity by increase of APEX protein.. Mutat Res.

[52] Vidal AE, Hickson ID, Boiteux S, Radicella JP (2001). Mechanism of stimulation of the DNA glycosylase activity of hOGG1 by the major human AP endonuclease: bypass of the AP lyase activity step.. Nucleic Acids Res.

[53] Sidorenko VS, Nevinsky GA, Zharkov DO (2007). Mechanism of interaction between human 8-oxoguanine-DNA glycosylase and AP endonuclease.. DNA Repair (Amst).

[54] Hill JW, Hazra TK, Izumi T, Mitra S (2001). Stimulation of human 8-oxoguanine-DNA glycosylase by AP-endonuclease: potential coordination of the initial steps in base excision repair.. Nucleic Acids Res.

[55] Kubota Y, Nash RA, Klungland A, Schar P, Barnes DE, Lindahl T (1996). Reconstitution of DNA base excision-repair with purified human proteins: interaction between DNA polymerase beta and the XRCC1 protein.. EMBO J.

[56] Caldecott KW, McKeown CK, Tucker JD, Ljungquist S, Thompson LH (1994). An interaction between the mammalian DNA repair protein XRCC1 and DNA ligase III.. Mol Cell Biol.

[57] Caldecott KW, Aoufouchi S, Johnson P, Shall S (1996). XRCC1 polypeptide interacts with DNA polymerase beta and possibly poly (ADP-ribose) polymerase, and DNA ligase III is a novel molecular 'nick-sensor' in vitro.. Nucleic Acids Res.

[58] Tebbs RS, Flannery ML, Meneses JJ, Hartmann A, Tucker JD, Thompson LH, Cleaver JE, Pedersen RA (1999). Requirement for the Xrcc1 DNA base excision repair gene during early mouse development.. Dev Biol.

[59] Pascucci B, Maga G, Hubscher U, Bjoras M, Seeberg E, Hickson ID, Villani G, Giordano C, Cellai L, Dogliotti E (2002). Reconstitution of the base excision repair pathway for 7,8-dihydro-8-oxoguanine with purified human proteins.. Nucleic Acids Res.

[60] Hashizume K, Hirasawa M, Imamura Y, Noda S, Shimizu T, Shinoda K, Kurihara T, Noda K, Ozawa Y, Ishida S, Miyake Y, Shirasawa T, Tsubota K (2008). Retinal dysfunction and progressive retinal cell death in SOD1-deficient mice.. Am J Pathol.

[61] Imamura Y, Noda S, Hashizume K, Shinoda K, Yamaguchi M, Uchiyama S, Shimizu T, Mizushima Y, Shirasawa T, Tsubota K (2006). Drusen, choroidal neovascularization, and retinal pigment epithelium dysfunction in SOD1-deficient mice: a model of age-related macular degeneration.. Proc Natl Acad Sci USA.

[62] Brennan LA, Kantorow M (2009). Mitochondrial function and redox control in the aging eye: Role of MsrA and other repair systems in cataract and macular degenerations.. Exp Eye Res.

